# Cost-effectiveness analysis of use of a polypill versus usual care or best practice for primary prevention in people at high risk of cardiovascular disease

**DOI:** 10.1371/journal.pone.0182625

**Published:** 2017-09-05

**Authors:** Sue Jowett, Pelham Barton, Andrea Roalfe, Kate Fletcher, F. D. Richard Hobbs, Richard J. McManus, Jonathan Mant

**Affiliations:** 1 Health Economics Unit, Institute of Applied Health Research, University of Birmingham, West Midlands, United Kingdom; 2 Primary Care Clinical Sciences, Institute of Applied Health Research, University of Birmingham, West Midlands, United Kingdom; 3 Nuffield Department of Primary Care Health Sciences, University of Oxford, Oxfordshire, United Kingdom; 4 Primary Care Unit, Department of Public Health & Primary Care, Strangeways Research Laboratory, University of Cambridge, Wort’s Causeway, Cambridge, Cambridgeshire, United Kingdom; Medical University Innsbruck, AUSTRIA

## Abstract

**Background:**

Clinical trials suggest that use of fixed-dose combination therapy (‘polypills’) can improve adherence to medication and control of risk factors of people at high risk of cardiovascular disease (CVD) compared to usual care, but cost-effectiveness is unknown.

**Objective:**

To determine whether a polypill is cost-effective compared to usual care and optimal guideline-recommended treatment for primary prevention in people already on statins and/or blood pressure lowering therapy.

**Methods:**

A Markov model was developed to perform a cost-utility analysis with a one year time cycle and a 10 year time horizon to compare the polypill with usual care and optimal implementation of NICE Guidelines, using patient level data from a retrospective cross-sectional study. The model was run for ten age (40 years+) and gender-specific sub-groups on treatment for raised CVD risk with no history of CVD. Published sources were used to estimate impact of different treatment strategies on risk of CVD events.

**Results:**

A polypill strategy was potentially cost-effective compared to other strategies for most sub-groups ranging from dominance to up to £18,811 per QALY depending on patient sub-group. Optimal implementation of guidelines was most cost-effective for women aged 40–49 and men aged 75+. Results were sensitive to polypill cost, and if the annual cost was less than £150, this approach was cost-effective compared to the other strategies.

**Conclusions:**

For most people already on treatment to modify CVD risk, a polypill strategy may be cost-effective compared with optimising treatment as per guidelines or their current care, as long as the polypill cost is sufficiently low.

## Introduction

Poor uptake of pharmacotherapy for people at high risk of cardiovascular disease, and lack of adherence in people who are prescribed drugs, has generated interest in the potential for fixed dose combination pills (‘polypills’).[1,2] These can bring about important reductions in blood pressure and LDL cholesterol,[3] and are associated with improved adherence to therapy.[4,5,6,7] However, despite evidence from trials demonstrating that polypills are largely safe and effective, [8] availability still remains limited compared with other disease areas [9]. Furthermore, no polypill for prevention of cardiovascular disease is currently licensed for use in the United Kingdom.

Previous cost-effectiveness analyses of polypills have primarily been concerned with treatment of secondary prevention patients [10,11], or, in primary prevention, comparing their use to no treatment, rather than to usual care or improved implementation of guidelines.[12,13] The aim of this study was to estimate the cost-effectiveness of a polypill strategy compared with current treatment or treatment as per guidelines for primary prevention for patients with known high cardiovascular risk who are already prescribed statins and/or blood pressure lowering therapy.

## Methods

A Markov cohort model developed in TreeAge Pro estimated cost-effectiveness of primary prevention with a polypill strategy compared with i) current therapy and ii) optimal therapy as per guidelines. The model considered patients aged 40 and over prescribed a statin and / or blood pressure lowering therapy with no history of cardiovascular disease. The model was run over a ten year time horizon with a one year cycle.

**Fig 1:**
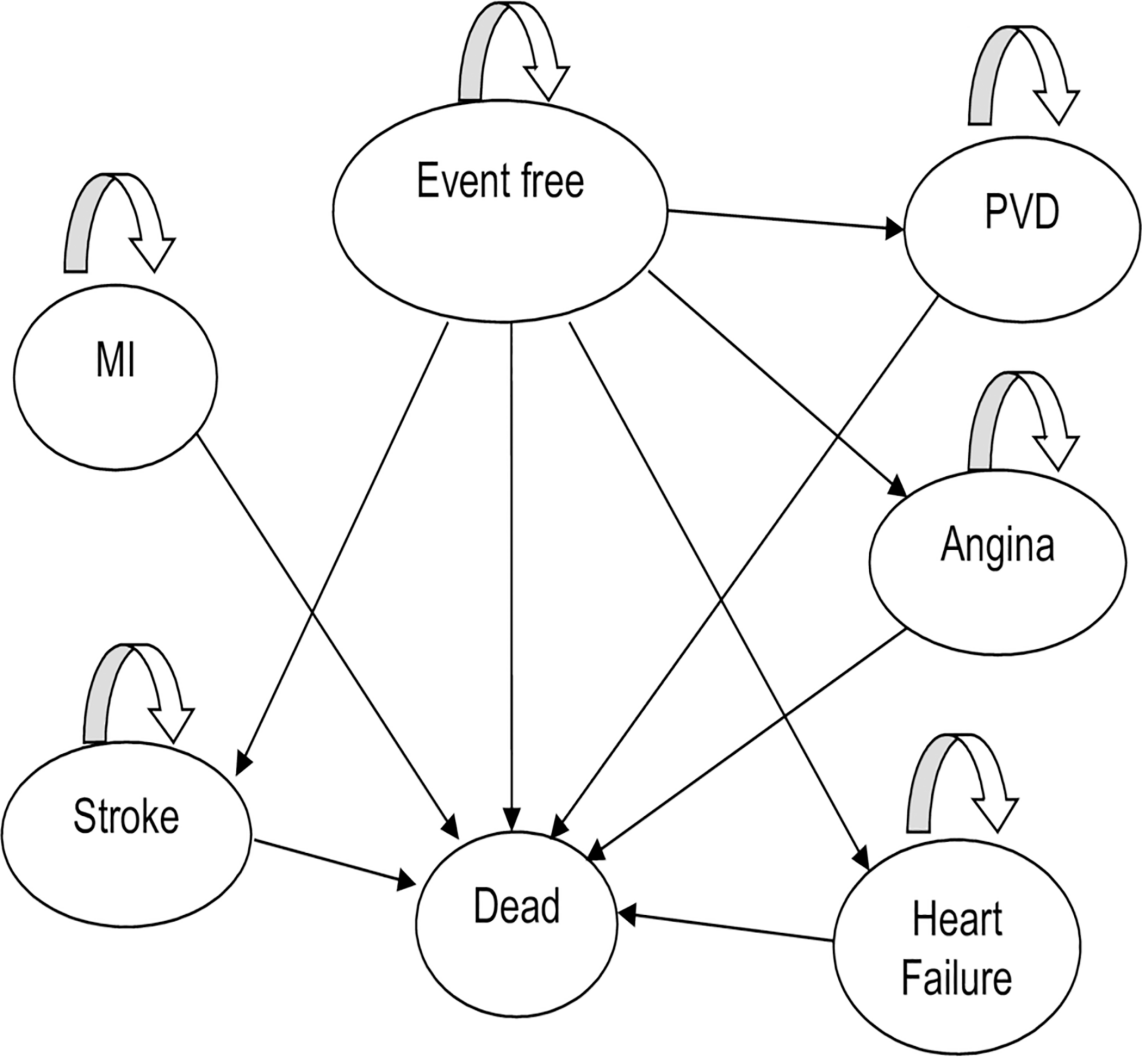
Model health states.

All patients started healthy and moved to other health states if they suffered stroke, myocardial infarction (MI), angina, heart failure or peripheral vascular disease (PVD) or died. Once a cardiovascular event occurred, they either died, or remained in this health state and incurred costs and a reduction in quality of life as assigned to that disease state until death (Fig 1).

### Study population

A cross sectional retrospective study of primary care medical records in 19 West Midland general practices in England provided data on risk factor profiles and current treatment.[14] Ten year cardiovascular risk was calculated using an updated Framingham equation.[15,16] The dataset was subdivided into ten age/gender subgroups (40–49, 50–59, 60–69, 70–74, 75 and over). Within each sub-group, eight treatment/cardiovascular risk strata were identified (S1 Table) that would be treated differently according to UK National Institute for Health and Care Excellence (NICE) guidelines.[17,18]

### Treatment strategies

Current treatment for each stratum was characterised by whether a statin was being taken, and if antihypertensives were being taken, the average number per strata.

The polypill strategy consisted of a pill a day containing a statin (40mg simvastatin) and three antihypertensives at half-dose (12.5mg hydrochlorothiazide, 5mg lisinopril, 2.5mg amlodipine).[19] As the patients were already taking medication, it was assumed the majority would take the polypill, with 16% discontinuing it (and therefore no longer incurring the cost of the polypill) and returning to their original treatment.[20] The polypill strategy was applied regardless of baseline cardiovascular risk or systolic blood pressure.

The guideline strategy assumed optimal treatment as per UK NICE guidelines.[17, 18] Statin therapy (simvastatin 40mg) was prescribed if cardiovascular risk was 20% or higher, and antihypertensives if blood pressure was greater than 140/90mm/Hg and cardiovascular risk was 20% or greater.[17] In those patients already on antihypertensives, it was assumed that additional drugs would be added in order to reach a target systolic blood pressure of 140mmHg, up to a maximum of three drugs. We estimated the additional number of antihypertensive drugs that would be required using the results of a meta-analysis.[21] For each subgroup we used the starting systolic blood pressure and the degree of blood pressure lowering required to determine through linear interpolation how many additional drugs would be needed.

### Impact of treatment

The baseline calculated 10 year cardiovascular risk was assumed to reflect benefit of current treatment (S1 Table), since the values of blood pressure and cholesterol in these patients reflected their current use of blood pressure lowering and lipid lowering drugs. For optimal guideline care, the impact of additional treatments was based on results of meta-analysis of randomised controlled trials (Table 1).[21, 22] We assumed 85% of people prescribed statins were fully compliant in taking their medication.[23] For the polypill strategy, treatment already being received was taken into account. If already on statins, then no additional effect from statins was applied. If antihypertensives were already being taken, the baseline systolic blood pressure and average number of drugs taken was used to determine the amount of BP lowering already being achieved, and what effect switching to three half dose drugs would have.[21] If switching to the polypill resulted in a lower dose of antihypertensives than current practice, risk estimates were adjusted accordingly.

**Table 1:** Summary of model inputs.

	Data	Sources
**Baseline mortality and risk of cardiovascular disease**		
Probability of stroke (10 years)	0.7–6.2%(age and sex dependent)	Calculated with Framingham [15,16] and risk factor profile based on patient level data
Probability of MI (10 years)	1.1–9.4% (age and sex dependent)	
Probability of angina (10 years)	1.5–13.3%(age and sex dependent)	
Probability of heart failure (10 years)	0.4–3.9%(age and sex dependent)	
Probability of PVD (10 years)	0.7–6.2% %(age and sex dependent)	
**Assumed distribution of possible CV events within 10 year CV risk**		
Stroke	16%	D’Agostino (2008) [16] Wood (2004) [24]
Myocardial infarction	24%	
Angina	34%	
Heart failure	10%	
PVD	16%	
**Risk reduction with statins**		
Stroke	0.80 (95% CI 0.73–0.86)	CTT (2005),[22] HPS (2002)[23]
MI, HF, angina	0.72 (95% CI 0.69–0.76)	CTT (2005), HPS (2002)
PVD	0.85 (95% CI 0.75–0.95)	HPS (2002)
**Probability of death from event**		
Fatal stroke	0.19	Ward (2007)[25]
Fatal MI	0.19–0.36 (Men)	Ward (2007)
	0.23–0.40 (Women)	
Fatal heart failure	0.17 (r = 68, n = 396)	Mehta (2009) [26]
SMR after stroke	2.72 (95% CI 2.59–2.85)	Bronnum-Hansen (2001) [27]
SMR after MI	2.68 (95% CI 2.48–2.91)	Bronnum-Hansen (2001) [28]
SMR after Heart Failure	2.17 (95% CI 1.96–2.41)	de Guili (2005) [29]
SMR after Angina	2.19 (95% CI 2.05–2.33)	NCGC [30]
SMR after PVD	2.44 (95% CI 1.59–3.74)	Leng (1996) [31]
**Reduction in blood pressure**		
Number of AHT drugs required to achieve target BP	0.60–1.52	Law (2009)[21]
**Reduction in CV risk with reduction in BP**		
**Polypill**		
CHD risk	10–52%	Law (2009)
Stroke risk	14–65%	Law (2009)
PVD risk	13–23%	Murabito (1997)[32]
	(Dependent on age, sex and risk group)	
**Treat to target**		
CHD risk	15–37%	Law (2009)
Stroke risk	20–47%	Law (2009)
PVD risk	13–32%	Murabito (1997)
	(Dependent on age, sex and risk group)	
Polypill adherence	84%	TIPS (2009)[20]
**Utilities**		
No cardiovascular event	(age and sex dependent)	General population utilities from EQ-5D (UK Tariff) (NCSR, 2006)[33]
Death	0	By definition
***Quality of life multipliers***		
Acute MI	0.76 (0.018)	Cooper (2008)[18], NICE (2014) [34]
Post MI	0.88 (0.018)	As above
Acute angina	0.77 (0.038)	As above
Post-acute angina	0.88 (0.018)	As above
Heart failure	0.68 (0.020)	As above
Stroke	0.63 (0.040)	As above
PVD	0.90 (0.020)	As above
**Costs**		
	£ per year	
Simvastatin 40mg	15.26	BNF March 2013 [35]
Amlodopine 5mg	12.13	BNF March 2013
Indapamide 2.5mg	11.87	BNF March 2013
Ramipril 5mg	18.13	BNF March 2013
Polypill	171	Assumed same price as Trinomia
	Unit cost £	
Blood test	15	Ward (2007)
GP visit	33	Curtis (2012) [36]
Practice nurse visit	11.25	Curtis (2012)
Acute events:	One-off cost £	
Stroke	11,020	Youman (2003) [37]
MI	5,487	Palmer (2002) [38]
Angina	3,292	Assumed 60% of MI cost
PVD	1,971	NHS Reference costs 2011/12 [39]
Heart failure	2,699	NHS Reference costs 2011/12
Long-term costs	£ per year	
Stroke	2721	Youman (2003)
MI	572	Cooper (2008) [18]
Angina	572	Cooper (2008)
PVD	302	Cooper (2008)
Heart failure	572	Cooper (2008)

SMR: Standardised Mortality Ratio; MI: Myocardial infarction; PVD: Peripheral Vascular Disease; CV: Cardiovascular

### Outcomes

Outcomes were measured in cardiovascular events and quality-adjusted life years (QALYs). A baseline utility value was applied depending upon age and gender.[33] When a cardiovascular event occurred, the health state value for that event was applied as a multiplier (Table 1). For consistency with other model-based analyses, utility values for CVD health states were obtained from previous UK NICE guidelines, where values were obtained through systematic review [18,34]. No reduction in quality of life was assumed for any drugs.[40]

Gender-specific life tables were used to determine the probability of death at different ages.[41] The risk of death was adjusted to ensure there was no double counting of cardiovascular death.[42] There was an increased risk of death once in a cardiovascular event health state.

### Costs

Costs assumed a UK NHS and personal social services perspective (Table 1). Polypill costs comprised: £171 (€192) a year for the pill, an initial GP visit and blood test in the first month, and an annual practice nurse visit and blood test thereafter. Due to the absence of a UK cost for a polypill, the cost was assumed to be in line that of an existing secondary prevention polypill (Trinomia^®^), which has a different composition. The cost was calculated using the mean of available prices and converted from US$ to UK£. In the current treatment and guideline strategies, the most commonly prescribed generic antihypertensive in each class (indapamide, amlodopine, ramipril) and the statin simvastatin were assumed.[35] Patients on antihypertensives were allocated four consultations (mix of GP and practice nurse) per year.[43] Two additional visits (one GP, one practice nurse) were included for guideline treatment in patients above target blood pressure.

### Analysis

An incremental cost-utility analysis was undertaken with a threshold of £20,000 per QALY taken to indicate cost-effectiveness. Future costs and QALYs were discounted at 3.5% per annum.[44] Costs were in UK pounds for 2011/12. Conversion into Euros was via the Purchasing Power Parity (PPP) Index for 2012, using a conversion rate of £1 to €1.125.[45] A half-cycle correction was applied to costs and effectiveness. We explored the impact of changing key parameters in a deterministic sensitivity analysis in a single age-sex stratum (men aged 60–69). Analysis of impact of price involved halving and doubling the price of a ‘polypill’ and reducing the cost to £57 (€64) a year, to reflect cost of individual generic agents.[35] The threshold price at which a polypill would become cost effective for each sub-group was determined. Where available, data were entered into the model as distributions so that a probabilistic sensitivity analysis could be undertaken. A log-normal distribution was used for all risk reductions and standardised mortality ratios after cardiovascular events, a beta distribution for cardiovascular event probabilities, risk of death from cardiovascular events and compliance with screening and a gamma distribution for acute and long-term costs. A Probabilistic Sensitivity Analysis (PSA) was run with 10,000 simulations and cost-effectiveness acceptability curves were produced (not shown) to provide information on the probability of interventions being cost-effective at different cost per QALY thresholds.

## Results

In the base-case analysis, the polypill strategy led to fewer CV events and was cost-effective over current practice and optimal treatment as per guidelines for men aged 50–74 and women over the age of 50. Subpopulation results varied from the polypill strategy being dominant (i.e. less costly and more effective), to Incremental Cost Effectiveness Ratios (ICERs) up to £18,811 (€21,162) per QALY gained (Tables 2 and 3). Optimal guideline care was dominant over the polypill for men aged over the age of 75 (but with very small differences in costs and QALYs), and most cost-effective in women aged 40–49.

**Table 2:** Results of the base-case analysis and probabilistic sensitivity analysis: Men.

Age group	Strategy	Mean cost (£)	Mean QALYs	Mean CV events	Incremental cost	Incremental QALYs	ICER (£ per QALY gained)	Probability of cost-effectiveness at £20,000/QALY	Polypill vs current practice
									ICER(£ per QALY gained)
40–49	Current practice	1,625	7.202	0.0956	0	0	-	0%	
	Optimal guideline care	1,634	7.216	0.0822	8	0.014	604	41%	
	Polypill	1,878	7.229	0.0683	244	0.014	18,057	59%	9,166
50–59	Current practice	2,008	6.740	0.1499	0	0	-	0%	
	Optimal guideline care	2,013	6.765	0.1290	5	0.025	182	0%	
	Polypill	2,136	6.784	0.1119	123	0.019	6,466	100%	2,897
60–69	Optimal guideline care	2,315	6.524	0.1714	0	0	-	0%	
	Current practice	2,343	6.477	0.2064	28	-0.047	Dominated	0%	
	Polypill	2,386	6.539	0.1592	71	0.015	4,791	100%	698
70–74	Optimal guideline care	2,429	5.916	0.1890	0	0	-	9%	
	Current practice	2,457	5.853	0.2334	28	-0.063	Dominated	0%	
	Polypill	2,459	5.922	0.1861	31	0.006	5,068	91%	33
	Optimal guideline care	2,320	4.782	0.1988	0	0	-	69%	
	Polypill	2,327	4.781	0.2005	7	-0.001	Dominated	31%	Dominant
75+	Current practice	2,395	4.692	0.2564	68	-0.089	Dominated	0%	

**Table 3:** Results of the base-case analysis and probabilistic sensitivity analysis: Women.

Age group	Strategy	Mean cost (£)	Mean QALYs	Mean CV events	Incremental cost	Incremental QALYs	ICER (£ per QALY gained)	Probability of cost-effectiveness at £20,000/QALY	Polypill vs current practice
									ICER (£ per QALY gained)
40–49	Current practice	1,325	7.077	0.0505	0	0	-	0%	
	Optimal guideline care	1,343	7.083	0.0446	18	0.006	2,994	94%	
	Polypill	1,671	7.093	0.0354	328	0.010	33,585	6%	21,798
50–59	Current practice	1,586	6.675	0.0894	0	0	-	0%	
	Optimal guideline care	1,599	6.688	0.0770	13	0.013	950	46%	
	Polypill	1,841	6.701	0.0644	243	0.013	18,811	54%	9,696
60–69	Current practice	1,805	6.513	0.1203	0	0	-	0%	
	Optimal guideline care	1,829	6.530	0.1060	23	0.018	1,304	2%	
	Polypill	1,994	6.546	0.0928	165	0.015	10,730	98%	5,667
70–74	Current practice	1,985	5.982	0.1492	0	0	-	0%	
	Optimal guideline care	2,042	6.009	0.1281	57	0.027	2,105	0%	
	Polypill	2,097	6.022	0.1170	55	0.013	4,245	100%	2,797
75+	Current practice	1,880	4.733	0.1644	0	0	-	0%	
	Optimal guideline care	1,947	4.774	0.1345	66	0.041	1,606	63%	
	Polypill	1,967	4.779	0.1303	20	0.005	4,131	37%	1,870

The probabilistic sensitivity analysis for all three treatment options showed that the polypill had a high probability (over 90%) of being cost-effective at a £20,000 (€22,500)/QALY threshold for men except for the youngest and oldest sub-groups. There was much more uncertainty in the results for women, with only the 60–69 and 70–74 sub-groups having a high probability of the polypill being the most cost-effective option. with considerable uncertainty around the results for those aged 50–59 (54% probability of being the most cost-effective option at £20,000/QALY). (Tables 2 and 3).

Deterministic sensitivity analyses for men aged 60–69 demonstrated that the superior cost effectiveness of a polypill over optimal guideline care over was robust to some underlying assumptions made in the model, with some key exceptions. Optimal guidelines became the most favourable strategy if take up of a polypill was low, if polypill was associated with a small reduction in quality of life, if polypill was less effective than assumed, and if the population was restricted to those with uncontrolled risk factors only (Table 4). The results were particularly sensitive to the cost of the polypill, with dominance achieved by halving the price or further reducing to the cost of the individual components. The superiority of the polypill compared with current practice in men aged 60–69 was sensitive to the cost of polypill, but robust to changes to the other assumptions (Table 5). Threshold analysis showed that the annual price of the polypill would need to be £152 (€171) or less to ensure cost-effectiveness at the £20,000 (€22,500)/QALY threshold for all sub-groups when compared with guidelines (Table 6).

**Table 4:** Deterministic sensitivity analysis results (men aged 60–69) for polypill strategy vs optimal guideline care.

	Cost difference vs. guidelines(£)	QALY difference vs. guidelines	Most CE strategy* and ICER (£/QALY) for polypill
**Base case**	71	0.015	Polypill (£4,791)
**Sensitivity analysis**			
Cost of polypill doubled	342	0.015	Guidelines (£76,849)
Cost of polypill halved	-462	0.015	Polypill dominates
Cost of polypill reduced to £57/year	-640	0.015	Polypill dominates
Decreased take up of polypill (25% take polypill)	95	-0.029	Guidelines dominates
Change cost of CV events			
increase by 30%	45	0.015	Polypill (£3,030)
decrease by 30%	97	0.015	Polypill (£6,553)
Quality of life reduction with polypill by 1%	71	-0.037	Guidelines dominates
Reduction in polypill effectiveness			
Antihypertensive effect reduced (statin effect fixed):			
50%	180	-0.004	Guidelines dominates
25%	126	0.006	Guidelines (£22,500)
Statins effect reduced (antihypertensive effect fixed) by 25%	95	0.010	Polypill (£9,397)
Antihypertensive and statin effect reduced by 25%	151	0.001	Guidelines (£228,788)
Increase costs of achieving optimal guideline care‡	-582	0.015	Polypill dominates
Study population restricted to people with uncontrolled risk factors at baseline†	-51	- 0.013	Guidelines (£3,952)**
Baseline CVD risk reduced by 30%	97	0.011	Polypill (£9,110)
Alternative time horizon			
20 years	49	0.048	Polypill (£1,011)
30 years	42	0.078	Polypill (£546)
Lifetime	40	0.084	Polypill (£473)

* CE at a £20,000/QALY gained threshold

** ICER is in the south-west quadrant and polypill is not CE as it is <£20,000/QALY

† i.e. ≥20% ten year cardiovascular risk and not on a statin, and/or with systolic blood pressure > 140 mmHg

‡ 4 additional (2 GP and 2 practice nurse) consultations per year over usual care, rather than 2 (1 of each).

**Table 5:** Deterministic sensitivity analysis results (men aged 60–69) for polypill strategy vs current practice.

	Cost difference vs. current practice	QALY difference vs. current practice	Most CE strategy* and ICER for polypill
**Base case**	43	0.062	Polypill (£698)
**Sensitivity analysis**			
Cost of polypill doubled	1,100	0.062	Polypill (£18,045)
Cost of polypill halved	-490	0.062	Polypill dominates
Cost of polypill reduced £57/year	-668	0.062	Polypill dominates
Decreased take up of polypill (25% take polypill)	67	0.018	Polypill (£3,702)
Change cost of CV events.			
CV events increase by 30%	-67	0.062	Polypill dominates
CV events decrease by 30%	153	0.062	Polypill (£2.490)
Quality of life reduction with polypill by 1%	43	0.001	Polypill (£4,475)
Reduction in polypill effectiveness			
Antihypertensive effect reduced (statin effect fixed)			
50%	152	0.043	Polypill (£3,517)
25%	98	0.052	Polypill (£1,865)
Statins effect reduced (antihypertensive effect fixed) by 25%	66	0.057	Polypill (£1,169)
Antihypertensive and statin effect reduced by 25%	122	0.047	Polypill (£2,582)
Study population restricted to people with uncontrolled risk factors at baseline†	-102	0.081	Polypill dominates
Baseline CVD risk reduced by 30%	143	0.045	Polypill (£3,206)
Alternative time horizon			
20 years	-5	0.190	Polypill dominates
30 years	12	0.293	Polypill (£40)
Lifetime	16	0.315	Polypill (£50)

* CE at a £20,000/QALY gained threshold

† i.e. >20% ten year cardiovascular risk and not on a statin, and/or with systolic blood pressure > 140 mmHg

**Table 6:** Optimal price of polypill.

Subgroup	Annual cost of polypill where the polypill is CE vs optimal guideline care (£)	Annual cost of polypill where the polypill is CE vs current practice (£)
**Male**		
40–49	175	215
50–59	210	285
60–69	207	361
70–74	187	408
75+	165	542
**Female**		
40–49	152	167
50–59	173	211
60–69	193	244
70–74	204	282
75+	185	324

(CE = <£20,000/QALY gained),. Base case price £365.25

## Discussion

Our base-case analysis suggests that using a polypill is more cost effective than usual practice for all age groups over 40 years apart from 40–49 year old women. The polypill is also cost-effective compared with optimal implementation of guidelines for most age and sex strata. Probabilistic sensitivity analysis showed that the superiority of polypill over usual care was very unlikely to be a chance finding, but there was more uncertainty over the comparison with optimal implementation of guidelines. This pattern was reflected in the deterministic sensitivity analysis which found that the superiority of polypill over usual care was robust to changing our assumptions, but not in comparison to optimal implementation of guidelines.

Switching to a polypill strategy may be a more cost effective way of improving cardiovascular prevention in people on treatment for raised cardiovascular risk than current practice or better implementation of guidelines in most patient sub-groups. However, this result was highly sensitive to cost of a polypill, take up of this treatment and potential effectiveness in reducing CV risk. At current individual drug prices, if a polypill cost £150 (€169) per year (i.e. a cost of 41p (€0.46) per pill), a polypill would be more cost effective than achieving optimal guideline care for all people over the age of 40 who are on treatment. Given that the costs of prescribing the individual components of the polypill are only around £57 (€64) per annum, this seems a feasible price not withstanding any technical difficulties of combining components in a single pill.

Previous cost effectiveness analyses have focussed on cost effectiveness of a polypill against no treatment, and found that this it is likely to be cost effective for primary prevention of high risk individuals in the developing world.[12, 13, 46]

Trials of using a polypill compared to usual care in people at high risk of cardiovascular disease have found better self-reported use of medication in the polypill arm,[5,6,7] and in one trial, this was also associated with better control of risk factors.[5] None of these trials included any intervention to enhance usual care.

The results need to be interpreted in the light of certain limitations. In a number of respects, the cost effectiveness of a polypill may have been under-estimated. The analysis was restricted to higher risk people already on treatment–inclusion of people not on medication would have increased the cost-effectiveness of polypill relative to current practice. Potential benefits of improved adherence to a polypill were not included.[5] It was assumed that 100% achievement of guideline targets is possible and indeed desirable.[47] However, this has probably not had a significant impact on overall results, since blood pressure target trials tend to show that mean blood pressure for the study population is below target, even if a substantial proportion of individuals have final blood pressure above target.[43,48,49] Thus, the impact of blood pressure lowering will have been over-estimated in some patients and under-estimated in others in the optimal implementation of guidelines strategy. The base-case analysis considered a 10-year time horizon as opposed to a life time horizon (which our sensitivity analysis showed tends to favour the polypill). This limited time horizon was chosen because of the complexities of estimating changes in risk factors (and therefore cardiovascular risk) over time. Finally, the risk of further events once someone had an initial cardiovascular event was not modelled, so potential benefits of treatments of secondary prevention were ignored.

Conversely, other assumptions favoured polypill. The separate drugs in the polypill were assumed to have additive effects. While one trial did find additive effects,[50] others have reported smaller combined effects.[3] The polypill was assumed to have no adverse effects on quality of life–sensitivity analysis showed that a small shift in this assumption would favour current practice. However, there is no empirical evidence of differences in quality of life between people on the polypill or usual care.[5] Optimal guideline care was based on guidelines in force in the UK up until 2014. Recent NICE guidelines have lowered the 10 year risk threshold for statin treatment from 20% to 10%.[34] This would result in a higher proportion of the study population being treated with statins in the optimal guideline implementation. This would have little effect on older age groups (see Table 1), but would result in increased effectiveness (and cost) of optimal guideline care in younger age groups. Finally, there are several other potential formulations of a polypill, which might have different effects on cardiovascular risk factors.[3]

## Conclusions

This analysis suggests that a polypill strategy may be a cost effective means to improve primary prevention in most people aged 50 and over with high cardiovascular risk on treatment, as long as the cost of a polypill is sufficiently low. If the cost of a polypill is lower than £150 (€169) per year, then this approach becomes cost effective for all sub-groups. However, despite the growing evidence base of the effectiveness of polypills,[3,5] such combinations are not yet generally available. This perhaps in part reflects reluctance of pharmaceutical companies to invest in multi-component pills and the hurdles posed by regulatory approval.[51] At the right price, a polypill strategy could be the most cost effective way of ensuring optimal cardiovascular risk reduction in people who are on treatment with antihypertensives or lipid lowering agents to lower their cardiovascular risk.

## Supporting information

S1 Table Baseline patient sub-group characteristics by age, sex and guideline category. (DOCX)

S1 File Technical appendix: Extended modelling methods. (DOCX)
