# Supplementary material for: Cost-effectiveness analysis of use of a polypill versus usual care or best practice for primary prevention in people at high risk of cardiovascular disease
Source: PLoS One. 2017 Sep 5;12(9):e0182625. doi: 10.1371/journal.pone.0182625 (PMC5584935; doi:10.1371/journal.pone.0182625)
Supplement: S1 Table — (DOCX) [file pone.0182625.s001.docx]

**S1 Table Baseline patient sub-group characteristics by age, sex and guideline category**

| **Men, age-group (mean age)[BP in mmHg]** | **Mean (SD) 10 year CV risk (%)** | **Mean (SD) Systolic BP [BP in mmHg]** | **Mean (SD) no. AHT drugs** | **Proportion at baseline (%)** |
| --- | --- | --- | --- | --- |
| **40-49 (45.0) n=273** |  |  |  |  |
| On statins, <=140 SBP | 8.4 (4.9) | 125.6 (10.1) | - | 15.4 |
| On statins, >140 SBP | 12.1 (6.4) | 151.6 (15.2) | - | 3.3 |
| On statins & AHT, <=140 SBP | 7.0 (3.2) | 128.2 (10.2) | 1.78 (0.70) | 14.7 |
| On statins & AHT, >140 SBP | 12.4 (9.5) | 150.7 (12.2) | 1.55 (0.83) | 10.6 |
| On AHT, <=140 SBP, 10y CVD <20% | 8.5 (3.6) | 131.4 (7.6) | 1.62 (0.74) | 33.3 |
| On AHT, <=140 SBP, 10y CVD 20%+ | 23.6 (1.0) | 139.5 (0.7) | 1.50 (0.81) | 0.7 |
| On AHT, >140 SBP, 10y CVD <20% | 11.7 (3.9) | 153.1 (11.1) | 1.58 (0.69) | 19.4 |
| On AHT, >140 SBP, 10y CVD 20%+ | 24.0 (4.8) | 168.6 (17.1) | 2.14 (0.69) | 2.6 |
| **50-59 (54.5), n=481** |  |  |  |  |
| On statins, <=140 SBP | 14.3 (7.1) | 127.6 (9.8) | - | 12.3 |
| On statins, >140 SBP | 22.1 (7.7) | 149.1 (6.3) | - | 3.9 |
| On statins & AHT, <=140 SBP | 12.9 (6.4) | 128.3 (8.9) | 1.72 (0.78) | 20.8 |
| On statins & AHT, >140 SBP | 19.8 (7.1) | 151.1 (10.5) | 1.95 (0.85) | 12.1 |
| On AHT, <=140 SBP, 10y CVD <20% | 12.5 (3.6) | 130.0 (7.9) | 1.63 (0.72) | 26.8 |
| On AHT, <=140 SBP, 10y CVD 20%+ | 24.2 (4.5) | 132.2 (6.5) | 1.56 (0.51) | 5.2 |
| On AHT, >140 SBP, 10y CVD <20% | 15.1 (2.8) | 149.3 (16.3) | 1.63 (0.80) | 11.6 |
| On AHT, >140 SBP, 10y CVD 20%+ | 25.3 (5.3) | 157.8 (14.3) | 1.66 (0.73) | 7.3 |
| **Men, age-group (mean age)** | **Mean 10 year CV risk (%)** | **Mean (SD) Systolic BP** | **Mean (SD) no. AHT drugs** | **Proportion at baseline (%)** |
| **60-69 (64.2), n=653** |  |  |  |  |
| On statins, <=140 SBP | 20.5 (8.7) | 128.9 (10.7) | - | 12.4 |
| On statins, >140 SBP | 25.8 (8.5) | 152.0 (9.1) | - | 4.4 |
| On statins & AHT, <=140 SBP | 19.1 (6.4) | 130.7 (8.1) | 1.81 (0.79) | 22.2 |
| On statins & AHT, >140 SBP | 26.5 (9.0) | 151.8 (12.3) | 1.77 (0.72) | 16.1 |
| On AHT, <=140 SBP, 10y CVD <20% | 15.8 (3.2) | 130.2 (9.3) | 1.65 (0.74) | 12.9 |
| On AHT, <=140 SBP, 10y CVD 20%+ | 25.5 (5.1) | 133.1 (7.4) | 1.58 (0.65) | 11.0 |
| On AHT, >140 SBP, 10y CVD <20% | 1.7 (1.9) | 145.3 (2.9) | 1.83 (0.76) | 4.5 |
| On AHT, >140 SBP, 10y CVD 20%+ | 29.1 (6.3) | 153.4 (10.1) | 1.8 (0.86) | 16.5 |
| **70-74 (71.8), n=266** |  |  |  |  |
| On statins, <=140 SBP | 24.5 (7.1) | 129.5 (9.3) | - | 8.7 |
| On statins, >140 SBP | 26.7 (3.4) | 148.0 (6.3) | - | 3.0 |
| On statins & AHT, <=140 SBP | 23.5 (5.5) | 130.0 (8.2) | 1.90 (0.74) | 18.8 |
| On statins & AHT, >140 SBP | 30.2 (8.2) | 150.3 (8.9) | 1.69 (0.75) | 24.4 |
| On AHT, <=140 SBP, 10y CVD <20% | 17.3 (2.1) | 122.3 (12.1) | 1.82 (0.87) | 4.1 |
| On AHT, <=140 SBP, 10y CVD 20%+ | 26.3 (5.7) | 132.4 (6.4) | 1.63 (0.73) | 20.3 |
| On AHT, >140 SBP, 10y CVD <20% | - | - | - | 0 |
| On AHT, >140 SBP, 10y CVD 20%+ | 31.2 (6.2) | 149.6 (8.1) | 1.69 (0.79) | 20.7 |

| **Men, age-group (mean age)** | **Mean 10 year CV risk (%)** | **Mean (SD) Systolic BP** | **Mean (SD) no. AHT drugs** | **Proportion at baseline (%)** |
| --- | --- | --- | --- | --- |
| **75+ (80.3), n=126** |  |  |  |  |
| On statins, <=140 SBP | 23.4 (3.1) | 126.7 (12.8) | - | 1.8 |
| On statins, >140 SBP | 33.2 (9.7) | 151.7 (8.9) | - | 2.9 |
| On statins & AHT, <=140 SBP | 25.5 (6.9) | 127.1 (10.3) | 1.59 (0.74) | 15.7 |
| On statins & AHT, >140 SBP | 34.8 (5.8) | 153.1 (11.3) | 2.00 (0.77) | 12.2 |
| On AHT, <=140 SBP, 10y CVD <20% | 18.7 (0.4) | 120.5 (27.6) | 3.00 (0.00) | 0.6 |
| On AHT, <=140 SBP, 10y CVD 20%+ | 31.3 (6.6) | 131.1 (8.8) | 1.77 (0.79) | 30.2 |
| On AHT, >140 SBP, 10y CVD <20% | - | - | - | 0 |
| On AHT, >140 SBP, 10y CVD 20%+ | 39.1 (7.8) | 152.5 (11.9) | 1.68 (0.72) | 36.6 |

SBP: Systolic Blood Pressure; AHT: Anti-Hypertensive Treatment; CV: Cardiovascular; CVD: Cardiovascular Disease

| **Women, age-group (mean age)** | **Mean (SD) 10 year CV risk** | **Mean (SD) Systolic BP** | **Mean (SD) no. AHT drugs** | **Proportion at baseline (%)** |
| --- | --- | --- | --- | --- |
| **40-49 (45.6), n=223** |  |  |  |  |
| On statins, <=140 SBP | 4.8 (2.8) | 122.1 (11.2) | - | 11.7 |
| On statins, >140 SBP | 4.6 (2.2) | 151.0 (9.5) | - | 1.3 |
| On statins & AHT, <=140 SBP | 4.6 (3.2) | 123.9 (10.4) | 1.56 (0.96) | 7.2 |
| On statins & AHT, >140 SBP | 5.8 (2.3) | 150.4 (6.9) | 1.60 (0.55) | 2.2 |
| On AHT, <=140 SBP, 10y CVD <20% | 4.4 (2.7) | 126.7 (10.2) | 1.34 (0.56) | 53.4 |
| On AHT, <=140 SBP, 10y CVD 20%+ | - | - | - | 0 |
| On AHT, >140 SBP, 10y CVD <20% | 8.4 (4.4) | 154.4 (13.7) | 1.54 (0.73) | 23.3 |
| On AHT, >140 SBP, 10y CVD 20%+ | 23.7 (4.3) | 153.0 (15.6) | 1.00 (0.00) | 0.9 |
| **50-59 (55.1), n=463** |  |  |  |  |
| On statins, <=140 SBP | 8.1 (4.1) | 125.8 (9.5) | - | 13.0 |
| On statins, >140 SBP | 12.8 (6.4) | 149.4 (7.7) | - | 3.5 |
| On statins & AHT, <=140 SBP | 7.9 (3.9) | 128.3 (9.2) | 1.58 (0.59) | 17.9 |
| On statins & AHT, >140 SBP | 10.9 (4.7) | 152.1 (11.8) | 1.68 (0.65) | 8.9 |
| On AHT, <=140 SBP, 10y CVD <20% | 7.4 (3.3) | 128.0 (9.4) | 1.59 (0.68) | 33.9 |
| On AHT, <=140 SBP, 10y CVD 20%+ | 23.4 (2.2) | 140.0 (0.0) | 1.00 (0.00) | 0.4 |
| On AHT, >140 SBP, 10y CVD <20% | 11.2 (3.8) | 152.2 (10.8) | 1.56 (0.67) | 19.4 |
| On AHT, >140 SBP, 10y CVD 20%+ | 25.2 (3.6) | 167.7 (12.7) | 1.71 (0.83) | 3.0 |

| **Women, age-group (mean age)** | **Mean (SD) 10 year CV risk** | **Mean (SD) Systolic BP** | **Mean (SD) no. AHT drugs** | **Proportion at baseline (%)** |
| --- | --- | --- | --- | --- |
| **60-69 (64.4), n=733** |  |  |  |  |
| On statins, <=140 SBP | 9.7 (4.7) | 127.9 (10.6) | - | 9.7 |
| On statins, >140 SBP | 15.3 (5.7) | 151.7 (12.1) | - | 4.8 |
| On statins & AHT, <=140 SBP | 11.1 (4.5) | 129.1 (9.1) | 1.67 (0.75) | 21.2 |
| On statins & AHT, >140 SBP | 15.9 (7.0) | 152.1 (10.9) | 1.70 (0.82) | 15.4 |
| On AHT, <=140 SBP, 10y CVD <20% | 10.9 (3.7) | 129.2 (10.4) | 1.71 (0.73) | 28.9 |
| On AHT, <=140 SBP, 10y CVD 20%+ | 23.1 (3.0) | 136.9 (2.9) | 1.92 (0.79) | 1.6 |
| On AHT, >140 SBP, 10y CVD <20% | 14.0 (2.9) | 149.9 (8.1) | 1.70 (0.72) | 13.8 |
| On AHT, >140 SBP, 10y CVD 20%+ | 23.7 (3.3) | 158.9 (16.9) | 1.65 (0.81) | 4.6 |
| **70-74 (71.9), n=353** |  |  |  |  |
| On statins, <=140 SBP | 13.0 (3.9) | 129.0 (9.1) | - | 6.0 |
| On statins, >140 SBP | 24.8 (8.1) | 157.3 (13.1) | - | 4.8 |
| On statins & AHT, <=140 SBP | 13.1 (4.4) | 131.6 (8.1) | 1.99 (0.84) | 21.8 |
| On statins & AHT, >140 SBP | 19.1 (6.5) | 149.9 (7.9) | 1.81 (0.76) | 17.8 |
| On AHT, <=140 SBP, 10y CVD <20% | 13.7 (3.6) | 131.4 (8.8) | 1.76 (0.74) | 20.4 |
| On AHT, <=140 SBP, 10y CVD 20%+ | 24.3 (2.7) | 132.6 (4.5) | 1.71 (0.76) | 2.0 |
| On AHT, >140 SBP, 10y CVD <20% | 16.5 (2.6) | 146.6 (4.1) | 1.69 (0.74) | 17.3 |
| On AHT, >140 SBP, 10y CVD 20%+ | 25.0 (5.0) | 156.0 (13.4) | 1.74 (0.66) | 9.9 |

| **Women, age-group (mean age)** | **Mean (SD) 10 year CV risk** | **Mean (SD) Systolic BP** | **Mean (SD) no. AHT drugs** | **Proportion at baseline (%)** |
| --- | --- | --- | --- | --- |
| **75+ (81.2), n=702** |  |  |  |  |
| On statins, <=140 SBP | 16.0 (7.6) | 125.6 (13.0) | - | 4.7 |
| On statins, >140 SBP | 20.3 (7.3) | 153.2 (10.7) | - | 3.0 |
| On statins & AHT, <=140 SBP | 15.6 (5.2) | 130.6 (9.5) | 1.90 (0.78) | 18.2 |
| On statins & AHT, >140 SBP | 22.6 (6.3) | 152.7 (11.1) | 1.86 (0.72) | 16.8 |
| On AHT, <=140 SBP, 10y CVD <20% | 16.1 (2.6) | 130.2 (8.4) | 1.64 (0.74) | 15.5 |
| On AHT, <=140 SBP, 10y CVD 20%+ | 24.2 (3.6) | 135.1 (6.2) | 1.62 (0.83) | 9.7 |
| On AHT, >140 SBP, 10y CVD <20% | 17.3 (1.6) | 146.2 (3.9) | 1.70 (0.69) | 5.7 |
| On AHT, >140 SBP, 10y CVD 20%+ | 27.8 (6.6) | 156.7 (14.9) | 1.71 (0.77) | 26.4 |

SBP: Systolic Blood Pressure; AHT: Anti-Hypertensive Treatment; CV: Cardiovascular; CVD: Cardiovascular Disease
