# Supplementary material for: Cost-effectiveness analysis of use of a polypill versus usual care or best practice for primary prevention in people at high risk of cardiovascular disease
Source: PLoS One. 2017 Sep 5;12(9):e0182625. doi: 10.1371/journal.pone.0182625 (PMC5584935; doi:10.1371/journal.pone.0182625)
Supplement: S1 File — (DOCX) [file pone.0182625.s002.docx]

**Technical appendix: Extended modelling methods**

*(note: reference numbers are from the main manuscript)*

**Methods**

A Markov cohort model developed in TreeAge Pro estimated cost-effectiveness of primary prevention with a polypill strategy compared with i) current therapy and ii) optimal therapy as per guidelines. The model considered patients aged 40 and over prescribed a statin and / or blood pressure lowering therapy with no history of cardiovascular disease. The model was run over a ten year time horizon with a one year time cycle.

All patients started in a well health state with no existing CVD, and moved to other health states if they suffered stroke, myocardial infarction (MI), angina, heart failure or peripheral vascular disease (PVD) or died. Once a cardiovascular event occurred, they either died, or remained in this health state and incurred costs and a reduction in quality of life as assigned to that disease state until death (Figure 1) .

**Fig1 Model health states**

**Study population**

A cross sectional retrospective study of primary care medical records in 19 West Midland general practices in England provided data on risk factor profiles and current treatment.[14] Data was available for 4273 patients (1799 men and 2474 women) on characteristics such as blood pressure and cholesterol levels and current treatment with statins and antihypertensives. Ten year cardiovascular risk was calculated for every patient using an updated Framingham equation.[15, 16] This ten year risk was then subdivided into five main possible CVD events (stroke, MI, angina, heart failure, PVD) using information from D’Agostino et al and other published data [12, 49]. This gave a ten year risk for all events for every patient, which was then converted into an annual risk.

The dataset was subdivided into ten age/gender subgroups (40-49, 50-59, 60-69, 70-74, 75 and over). Within each sub-group, eight treatment/cardiovascular risk strata were identified (S1 Table) that may be treated differently according to UK National Institute for Health and Care Excellence (NICE) guidelines.[17,18,34] The strata were distinguished by combinations of current treatment (statins and/or antihypertensives), whether systolic blood pressure (SBP) was above or below 140mm/Hg and if 10 year CVD risk was above or below 20% (in those currently not receiving statins). For each sub-group stratum the mean 10 year CVD risk, mean 10 year risks for each type of CVD event, systolic blood pressure, and number of antihypertensive drugs was calculated from the patient-level data. This acted as baseline data for current treatment in the model, and the average characteristics and probabilities of CVD events in each stratum for each sub-group were used in the model.

**Treatment strategies**

Current treatment for each stratum was characterised by whether a statin was being taken, and if antihypertensives were being taken, the average number per strata. The polypill strategy consisted of a pill a day containing a statin (40mg simvastatin) and three antihypertensives at half-dose (12.5mg hydrochlorothiazide, 5mg lisinopril, 2.5mg amlodipine).[19] As the patients were already taking medication, it was assumed the majority would take the polypill, with 16% discontinuing it (and therefore no longer incurring the cost of the polypill) and returning to their original treatment.[20] The polypill strategy was applied regardless of baseline cardiovascular risk or systolic blood pressure.

The guideline strategy assumed optimal treatment as per UK NICE guidelines.[17, 18] Statin therapy (simvastatin 40mg) was prescribed if cardiovascular risk was 20% or higher, and antihypertensives if blood pressure was greater than 140/90mm Hg and cardiovascular risk was 20% or greater.[17] In those patients already on antihypertensives but with a systolic blood pressure of greater than 140mm Hg, it was assumed that additional drugs would be added in order to reach a target systolic blood pressure of 140mmHg, up to a maximum of three drugs. We estimated the additional number of antihypertensive drugs that would be required using the results of a meta-analysis.[21] For each subgroup we used the starting systolic blood pressure and the degree of blood pressure lowering required to determine, through linear interpolation, how many additional drugs would be needed.

**Impact of treatment**

The baseline calculated 10 year cardiovascular risk was assumed to reflect benefit of current treatment (S1 Table), since the values of blood pressure and cholesterol in these patients reflected their current use of blood pressure lowering and lipid lowering drugs. For optimal guideline care, the impact of additional treatments on risk of CVD was based on results of meta-analysis of randomised controlled trials (Table 1).[21,22] We assumed that whilst all patients prescribed statins received a prescription (and therefore costs were incurred by the health service), only 85% of people prescribed statins were fully compliant in taking their medication.[23] For the polypill strategy, treatment already being received was taken into account. If already on statins, then no additional effect from statins was applied. If antihypertensives were already being taken, the baseline systolic blood pressure and average number of drugs taken was used to determine the amount of BP lowering already being achieved, and what effect switching to three half dose drugs would have.[21] If switching to the polypill resulted in a lower dose of antihypertensives than current practice, risk estimates were adjusted accordingly i.e. patients were at greater risk of CVD.

**Table 1 Summary of Model inputs**

|  | **Data** | **Sources** |
| --- | --- | --- |
| **Baseline mortality and risk of cardiovascular disease** | | |
| Probability of stroke  (10 years) | 0.7-6.2%  (age and sex dependent) | Calculated with Framingham [15,16] and risk factor profile based on patient level data |
| Probability of MI  (10 years) | 1.1-9.4%  (age and sex dependent) |  |
| Probability of angina  (10 years) | 1.5-13.3%  (age and sex dependent) |  |
| Probability of heart failure (10 years) | 0.4-3.9%  (age and sex dependent) |  |
| Probability of PVD  (10 years) | 0.7-6.2% %  (age and sex dependent) |  |
| **Assumed distribution of possible CV events within 10 year CV risk** | | |
| Stroke | 16% | D’Agostino (2008) [16]  Wood (2004) [24] |
| Myocardial infarction | 24% |  |
| Angina | 34% |  |
| Heart failure | 10% |  |
| PVD | 16% |  |
|  |  |  |
| **Risk reduction with statins** | | |
| Stroke | 0.80 (95% CI 0.73-0.86) | CTT (2005),[22] HPS (2002)[23] |
| MI, HF, angina | 0.72 (95% CI 0.69-0.76) | CTT (2005), HPS (2002) |
| PVD | 0.85 (95% CI 0.75-0.95) | HPS (2002) |
| **Probability of death from event** | | |
| Fatal stroke | 0.19 | Ward (2007)[25] |
| Fatal MI | 0.19-0.36 (Men)  0.23-0.40 (Women) | Ward (2007) |
| Fatal heart failure | 0.17 (r=68, n=396) | Mehta (2009) [26] |
| SMR after stroke | 2.72 (95% CI 2.59-2.85) | Bronnum-Hansen (2001) [27] |
| SMR after MI | 2.68 (95% CI 2.48-2.91) | Bronnum-Hansen (2001) [28] |
| SMR after Heart Failure | 2.17 (95% CI 1.96-2.41) | de Guili (2005) [29] |
| SMR after Angina | 2.19 (95% CI 2.05-2.33) | NCGC [30] |
| SMR after PVD | 2.44 (95% CI 1.59-3.74) | Leng (1996) [31] |
| **Reduction in blood pressure** | | |
| Number of AHT drugs required to achieve target BP | 0.60-1.52 | Law (2009)[21] |
| **Reduction in CV risk with reduction in BP** | | |
| **Polypill**  CHD risk  Stroke risk  PVD risk | 10-52%  14-65%  13-23%  (Dependent on age, sex and risk group) | Law (2009)  Law (2009)  Murabito (1997)[32] |
| **Treat to target**  CHD risk  Stroke risk  PVD risk | 15-37%  20-47%  13-32%  (Dependent on age, sex and risk group) | Law (2009)  Law (2009)  Murabito (1997) |
| Polypill adherence | 84% | TIPS (2009)[20] |
| **Utilities** |  |  |
| No cardiovascular event  Death  ***Quality of life multipliers***  Acute MI  Post MI  Acute angina  Post-acute angina  Heart failure  Stroke  PVD | (age and sex dependent)  0  0.76 (0.018)  0.88 (0.018)  0.77 (0.038)  0.88 (0.018)  0.68 (0.020)  0.63 (0.040)  0.90 (0.020) | General population utilities from EQ-5D (UK Tariff) (NCSR, 2006)[33]  By definition  Cooper (2008)[18], NICE (2014) [34]  As above  As above  As above  As above  As above  As above |
| **Costs** |  |  |
| Simvastatin 40mg  Amlodopine 5mg  Indapamide 2.5mg  Ramipril 5mg  Polypill  Blood test  GP visit  Practice nurse visit  Acute events:  Stroke  MI  Angina  PVD  Heart failure  Long-term costs:  Stroke  MI  Angina  PVD  Heart failure | £ per year  15.26  12.13  11.87  18.13  171  Unit cost  15  33  11.25  One-off cost £  11,020  5,487  3,292  1,971  2,699  £ per year  2721  572  572  302  572 | BNF March 2013 [35]  BNF March 2013  BNF March 2013  BNF March 2013  Assumed same price as Trinomia^®^  Ward (2007)  Curtis (2012) [36]  Curtis (2012)  Youman (2003) [37]  Palmer (2002) [38]  Assumed 60% of MI cost  NHS Reference costs 2011/12 [39]  Youman (2003)  Cooper (2008) [18]  Cooper (2008)  Cooper (2008)  Cooper (2008) |

SMR: Standardised Mortality Ratio; MI: Myocardial infarction; PVD: Peripheral Vascular Disease; CV: Cardiovascular

**Outcomes**

Outcomes were measured in cardiovascular events and quality-adjusted life years (QALYs). A baseline average utility value was applied depending upon age and gender, using values calculated from the general population in the UK.[33] When a cardiovascular event occurred, the health state value for that event was applied as a multipler (Table 1). For consistency with other model-based analyses, utility values for CVD health states were obtained from previous UK NICE guidelines, where values were obtained through systematic review [18,34]. As data on utility values for cardiovascular disease do not come from one data source, due to the lack of availability of this data, it is inevitable that values are sourced from many different studies, conducted in different settings, and eliciting preferences using different methods. No reduction in quality of life was assumed for any drugs.[40]

Gender-specific life tables were used to determine the probability of death at different ages.[41] The risk of death was adjusted to ensure there was no double counting of cardiovascular death.[42] There was an increased risk of death once in a cardiovascular event health state, with standardised mortality ratios applied to the risk of death.

**Costs**

Costs assumed a UK NHS and personal social services perspective (Table 1). Polypill costs comprised: £171 (€192) a year for the pill, an initial GP visit and blood test in the first month, and an annual practice nurse visit and blood test thereafter. Due to the absence of a UK cost for a polypill, the cost was assumed to be in line that of an existing secondary prevention polypill (Trinomia^®^). This drug has a different composition and contains aspirin, a statin and ramipiril. The cost was calculated using the mean of two available prices and converted from US$ to UK£. In the current treatment and guideline strategies, the most commonly prescribed generic antihypertensive in each class (indapamide, amlodopine, ramipril) and the statin simvastatin were assumed.[35] Patients on antihypertensives were allocated four consultations (mix of GP and practice nurse) per year, for monitoring of blood pressure.[43] Two additional visits (one GP, one practice nurse) were included for guideline treatment in patients above target blood pressure. CVD events were comprised of a one-off acute care cost and an annual cost representing long-term post-event care. Costs were sourced from standard UK reference costs [35,36,39], previously published decision models conducted for UK guideline development [18, 34] and costing studies [37].

**Analysis**

An incremental cost-utility analysis was undertaken with a threshold of £20,000 per QALY taken to indicate cost-effectiveness. Future costs and QALYs were discounted at 3.5% per annum.[440] Costs were in UK pounds for 2011/12. Conversion into Euros was via the Purchasing Power Parity (PPP) Index for 2012, using a conversion rate of £1 to €1.125.[45] A half-cycle correction was applied to costs and effectiveness. We explored the impact of changing key parameters in a deterministic sensitivity analysis in a single age-sex stratum (men aged 60-69). . Analysis of impact of price involved halving and doubling the price of a ‘polypill’ and reducing the cost to £57 (€64) a year, to reflect cost of individual generic agents.[35] The threshold price at which a polypill would become cost effective for each sub-group was determined. Where available, data were entered into the model as distributions so that a probabilistic sensitivity analysis could be undertaken. A log-normal distribution was used for all risk reductions and standardised mortality ratios after cardiovascular events, a beta distribution for cardiovascular event probabilities, risk of death from cardiovascular events and compliance with screening and a gamma distribution for acute and long-term costs. A Probabilistic Sensitivity Analysis (PSA) was run with 10,000 simulations and cost-effectiveness acceptability curves were produced (not shown) to provide information on the probability of interventions being cost-effective at different cost per QALY thresholds.
